# Effekte von präoperativer neurobiologischer Edukation auf das postoperative Outcome: Ein systematisches Review

**DOI:** 10.1007/s00482-021-00608-8

**Published:** 2022-01-05

**Authors:** Karolin von Korn, Thomas Weiss, Harry von Piekartz

**Affiliations:** 1 Lubinus Aktiv GmbH, Steenbeker Weg 33, 24106 Kiel, Deutschland; 2 Fakultät Wirtschafts- und Sozialwissenschaften, Physiotherapie und Rehabilitationswissenschaften, Hochschule Osnabrück, Caprivistr. 30a, 49076 Osnabrück, Deutschland; 3 Lehrstuhl für Klinische Psychologie, Friedrich-Schiller-Universität Jena, Am Steiger 3, Haus 1, 07743 Jena, Deutschland

**Keywords:** Präoperative neurobiologische Edukation, Wirbelsäulenoperation, Angstreduktion, Katastrophisieren, Postoperative Chronifizierung, Preoperative pain neuroscience education, Spinal surgery, Anxiety reduction, Catastrophizing, Postoperative chronification

## Abstract

**Hintergrund:**

Schmerz hat einen entscheidenden Einfluss auf die humane Lebensqualität. Allein eine Wissensvergrößerung über neurobiologische Vorgänge kann das subjektive Schmerzempfinden sowie psychometrische Variablen positiv beeinflussen. Es gibt verschiedene Formen der präoperativen Patientenedukation, welche u. a. zum Ziel haben, den postoperativen Schmerz zu erklären. Laut der aktuellen Literatur liegt einer präoperativen biomedizinischen Edukation eine geringe Evidenz zugrunde. Sie kann das präoperative Angst- und Stresslevel der Patienten steigern, was sich negativ auf das postoperative Outcome auswirkt. Im Gegensatz zur biomedizinischen Edukation betrachtet das neurobiologische Verständnis den postoperativen Schmerz unter den Gesichtspunkten der Plastizität des Nervensystems und bezieht Sensibilisierungsprozesse im zentralen und peripheren Nervensystem mit ein.

**Ziel:**

Systematische Untersuchung von Kurz- und Langzeiteffekten einer neurobiologischen (Schmerz‑)Edukation bei Patienten vor einer Wirbelsäulenoperation

**Material und Methoden:**

Bei der Literaturrecherche wurde nach dem PI(C)O(Population Intervention Comparison Outcome)‑Schema in den medizinischen Datenbanken gesucht. 83 Artikel kamen in die engere Auswahl. Entsprechend den Ein- und Ausschlusskriterien konnten letztendlich neun Artikel eingeschlossen werden.

**Ergebnisse:**

Durch eine präoperative neurobiologische (Schmerz‑)Edukation können postoperative Katastrophisierungstendenzen sowie die postoperative Kinesiophobie positiv beeinflusst werden. Keinen Einfluss hat eine präoperative neurobiologische Edukation auf postoperativen Schmerz und Funktion. Inkonsistenz besteht bislang in der Herangehensweise der PNE (Pain Neuroscience Education).

**Schlussfolgerung:**

Eine präoperative Reduktion von Angst und schmerzaufrechterhaltenden Faktoren v. a. auf psychologischer und sozialer Ebene hat einen positiven Effekt auf die postoperative subjektive Schmerzbewertung, was sich in einer Reduktion von Angst, Katastrophisierungstendenzen und einer geringeren Inanspruchnahme von postoperativen Leistungen im Gesundheitswesen widerspiegelt.

## Abstract (en)

**Background:**

Pain may have a crucial impact on human quality of life. An increase in knowledge about neurobiological and neuroscientific processes alone can positively influence the subjective perception of pain as well as psychometric variables. There are different forms of preoperative patient education with the aim to explain postoperative pain. Based on current literature, preoperative biomedical education has a low level of evidence. It can increase the preoperative anxiety and stress level of patients, which has a negative impact on the postoperative outcome. In contrast, the neuroscientific understanding considers postoperative pain from the viewpoints of the plasticity of the nervous system and involves sensitizational processes in the central and peripheral nervous systems.

**Purpose:**

To systematically investigate short- and long-term effects of pain neuroscience education (PNE) in patients before spine surgery.

**Materials and methods:**

The literature search involved a search of medical databases according to the PI(C)O scheme, and 83 articles were shortlisted. Nine articles that met the inclusion and exclusion criteria were finally included.

**Results:**

Preoperative pain neuroscience education can positively influence postoperative catastrophizing tendencies as well as postoperative kinesiophobia but has no influence on postoperative pain and function.

**Conclusion:**

Preoperative reduction of anxiety and pain-maintaining factors mainly on the psychological and social level may have a positive effect on postoperative subjective pain evaluation, which is reflected in a reduction of anxiety, catastrophizing tendencies, and a lower utilization of postoperative health care services.

Trotz erfolgreicher Operation fallen Patienten postoperativ regelmäßig durch kinesiophobes und katastrophisierendes Verhalten sowie anhaltenden Schmerz auf. Es gibt Hinweise, wonach eine ausschließliche biomedizinische Aufklärung (BMA) des OP-Prozederes diese Verhaltensentwicklung begünstigen kann. Im Gegensatz zu einer BMA wird durch eine neurobiologische (Schmerz‑)Edukation (PNE) das subjektive Schmerzempfinden vor einem neurobiologischen und neurophysiologischen Hintergrund erklärt. In diesem Review wird der Frage nachgegangen, ob eine zusätzliche perioperative PNE einen positiven Einfluss auf die postoperativen Variablen, insbesondere Kinesiophobie, Katastrophisieren und Schmerz, hat [2].

## Einleitung

Anhaltende Schmerzen sind für den Betroffenen sehr beeinträchtigend und haben häufig negative Auswirkungen auf soziale, familiäre Kontakte und den beruflichen Alltag. Trotz einer sich stetig verbessernden Qualität der OP-Techniken konnte gleichzeitig keine eindeutige physische und mentale postoperative Gesundheitsverbesserung der Patienten erzielt werden [1], was hohe Chronifizierungsraten postoperativer Schmerzen impliziert [8, 13, 14]. Eine Vielzahl von „randomised controlled trials“ (RCT) zeigte bereits einen positiven Effekt durch eine neurobiologische Schmerzedukation („pain neuroscience education“ [PNE]) bei Patienten mit chronischen lumbalen Schmerzen [13, 24]. Diese Überlegungen lassen die Fragestellung zu, ob eine präoperative PNE einen positiven Einfluss auf das postoperative Outcome und damit auf geringere postoperative Schmerzchronifizierungsraten hat.

### Chronifizierungsgründe

Nicht selten entwickelt sich eine Schmerzchronifizierung nach einer Operation.

Neuere Untersuchungen von verschiedenen Autoren zeigen, dass bei etwa 40 % aller Patienten, die in den USA aufgrund von degenerativen Veränderungen der Wirbelsäule operiert werden, der Schmerz weit über den normalen Heilungsverlauf einer verletzten Struktur hinaus andauert – sie chronifizieren. Zudem weisen Patienten nach einer OP deutliche funktionelle Defizite auf [1, 8, 13, 14].

Die Entwicklung einer (postoperativen) Chronifizierung ist nicht von objektivierbaren Daten (Geschlecht, Familienstand, Ausbildungsstatus) abhängig [7, 30], sondern wird neben einer insuffizienten Stellung der OP-Indikation und einem bereits präoperativ bestehenden chronischen Schmerz insbesondere durch neuroplastische Veränderungen (periphere Sensibilisierung, zentrale Sensibilisierung, deszendierende Modulation) bedingt. Infolgedessen treten häufig maladaptive Copingstrategien, ein Angst-Vermeidungs-Verhalten, Katastrophisierungsgedanken und negative Emotionen, insbesondere Angst, auf. Schmerzen, welche als bedrohlich empfunden werden, verhindern die Rückkehr zu einer normalen Aktivität [28].

Menschen mit einem ausgeprägten Angst-Vermeidungs-Verhalten (AVV) und Neigungen zur Schmerzkatastrophisierung chronifizieren häufiger [1, 18, 20, 27, 29]. Emotionen und die daraus resultierenden physiologischen Reaktionen eines Menschen können durch Gedanken und Überzeugungen positiv oder negativ beeinflusst werden [27].

Zu den allgemeinen Hauptrisikofaktoren für eine Chronifizierung von Rückenschmerzen gehören der subjektive Umgang mit Arbeitsplatzkonflikten, soziale Isolation, Depressionen, negative Emotionen und Katastrophisierungsgedanken, welche ggf. auch auf eine mangelhafte Aufklärung zurückzuführen sind [7].

### Neurobiologische Schmerzedukation (PNE)

Neuere Ansätze in der Behandlung chronischer Schmerzsyndrome haben eine neurobiologische Edukation als Haupttherapiebaustein integriert. Ziel dieser Edukation ist es, dem Patienten ein umfassendes Wissen über die neurobiologischen und neurophysiologischen Zusammenhänge des subjektiven Schmerzempfindens zu vermitteln und somit die bedrohende Wirkung des Schmerzes zu reduzieren.

Eine Übersicht der „key points“ der biomedizinischen sowie der neurobiologischen Edukation ist in Tab. 1 beschrieben, generelle Eigenschaften bezüglich der Inhalte einer PNE finden sich in Tab. 2.

**Table 1:** 

	Biomedizinische Edukation	Neurobiologische Edukation
Hauptpunkte	Erklärung des OP-Prozederes	Physiologie des ZNS und PNS
Herangehensweise	Biomedizinische/biomechanische Herangehensweise	Biopsychosoziale Herangehensweise
Evidenz	Geringe Evidenz [14]	Erste evidenzbasierte Ergebnisse weisen einen guten Effekt auf
Schmerzspezifizierung	Geringe Schmerzspezifizierung [13] Erklärung von (andauerndem) Schmerz immer als Zeichen einer Gewebeschädigung [16]	Betrachtung von (andauerndem) Schmerz unter den Gesichtspunkten der Plastizität des Nervensystems und Sensibilisierungsprozesse im ZNS und PNS. Ziel: Reduktion der Angst vor dem Schmerz [13, 14, 16]
Postulierter Effekt der präoperativen Edukation	Möglicherweise kann sich das präoperative Angst- und Stresslevel der Patienten steigern, was sich negativ auf das postoperative Outcome auswirkt [14]	Reduktion des präoperativen Angst- und Stresslevels

*ZNS* zentrales Nervensystem, *PNS* peripheres Nervensystem

**Table 2:** 

Inhaltspunkte der PNE
1. Bestärkung der Entscheidung zu einer Operation
2. Anatomie und Physiologie des Nervensystems
3. Das Nervensystem und der Rücken
4. Das Nervensystem im Kontext einer OP
5. Schmerzregulierung im zentralen Nervensystem
6. Genesung nach der Operation

*PNE* Pain Neuroscience Education

In einem RCT von Louw et al. (2014) konnte bereits ein positiver Effekt in Bezug auf den Schmerz, das Katastrophisieren und die Funktion/Beweglichkeit durch eine PNE bei Patienten mit chronischen lumbalen Schmerzen im 1‑Jahres-Follow-up zeigen [13, 24]. Schmerz als solcher hat primär die Funktion der „Warnung vor einer Bedrohung“ und stellt somit eine Schutzfunktion vor einer potenziellen Gefahr dar. Allein das Verstehen des Schmerzes führt zu einer Reduktion der subjektiv empfundenen Bedrohung durch den Schmerz [3, 8]. Diese Überlegungen lassen die Fragestellung zu, welchen Effekt eine präoperative PNE auf das postoperative Outcome hätte.

Das Ziel dieses systematischen Reviews ist, den Effekt einer präoperativen neurophysiologischen Schmerzedukation auf das postoperative Outcome sowie die unterschiedlichen Herangehensweisen (bzgl. Dauer und Setting) dieser Intervention zu analysieren.

Die *primäre Forschungsfrage* hierzu lautet: Welchen Effekt zeigt eine präoperative neurophysiologische Schmerzedukation auf das postoperative Outcome?

Die *sekundäre Forschungsfrage* lautet: Welche Herangehensweisen wählen die Autoren zur präoperativen neurophysiologischen Schmerzedukation?

## Methode

Die medizinische Datenbank PubMed wurde nach englischen und deutschen Artikeln durchsucht. Bei der Suche wurde nach dem PI(C)O-Prinzip vorgegangen und die Suchbegriffe aus Tab. 3 verwendet. Da die einfache Suche in „All Fields“ sehr unspezifisch war und sehr hohe Trefferzahlen zur Folge hatte, wurde anschließend mit der MeSH(Medical Subject Headings)-Term-Funktion und in der Kategorie „Title/Abstract“ gesucht.

**Table 3:** 

Suchbegriffe	Funktion	Treffer
Chronic pain	MeSH Terms	14.774
Low back pain	MeSH Terms	21.965
Pain neuroscience education	MeSH Terms	248
Preoperative pain neuroscience education	MeSH Terms	12
Pain	Title/Abstract	631.164
Disability	Title/Abstract	158.684
Kinesiophobia	Title/Abstract	873
Catastrophizing	Title/Abstract	3197
Central sensitization	Title/Abstract	2876
Central sensitization	MeSH Terms	634

*MeSH* Medical Subject Headings

### Schritte der systematischen Literaturrecherche

Die Verknüpfung wurde durch die Boulschen Operatoren „AND“ und „OR“ wie folgt vorgenommen:

#### 1. Verknüpfung.

– 75 Treffer:

((((((chronic pain[MeSH Terms]) AND (pain neuroscience education[MeSH Terms])) AND (pain[Title/Abstract])) OR (disability[Title/Abstract])) AND (kinesiophobia[Title/Abstract])) OR (catastrophizing[Title/Abstract])) AND (central sensitization[Title/Abstract])

#### 2. Verknüpfung.

– 2 Treffer:

(((chronic pain[MeSH Terms]) AND (pain neuroscience education[MeSH Terms])) AND (pain[MeSH Terms])) AND (central sensitization[MeSH Terms])

#### 3. Verknüpfung.

– 6 Treffer:

(((((((low back pain[MeSH Terms]) AND (preoperative pain neuroscience education)) OR (preoperative management)) AND (pain[Title/Abstract])) OR (disability[Title/Abstract])) AND (kinesiophobia[Title/Abstract])) AND (catastrophizing[Title/Abstract])) AND (central sensitization[Title/Abstract])

Auf Basis der Abstracts wurden 83 Artikel entsprechend den unten aufgeführten Ein- und Ausschlusskriterien gescreent.

Zur Vertiefung der Thematik wurde bei google.scholar.com und scinos.de nach Artikeln gesucht. Auch wurden Verweise in den vorliegenden Artikeln und Studien auf die dort verwendeten Quellenangaben aufgenommen.

### Einschlusskriterien

Die Artikel wurden eingeschlossen, wenn sie ab dem 01.01.2011 erschienen sind, ein Evidenzlevel 1, 2, 3 oder 4 erfüllten, eine biomedizinische und/oder eine neurobiologische Edukation bei Patienten vor einer Operation durchgeführt wurde und zu den untersuchten Outcomeparametern die medizinisch entstehenden Kosten, Schmerz, Funktion, Katastrophisierung und/oder Kinesiophobie zählten.

Ausgeschlossen wurden die Artikel bei einem Erscheinungsdatum vor 01.01.2011, mit einem Evidenzlevel 5, wenn keine präoperative Edukation durchgeführt wurde sowie bei anderen als den oben erwähnten Outcomeparametern.

## Ergebnisse

Nach dem Screening der Abstracts wurden neun Studien [10, 11, 13–16, 19, 25, 26] eingeschlossen, welche an *n* = 360 Probanden den zusätzlichen Effekt von verschiedenen präoperativen Edukationen auf das postoperative Outcome untersuchten. Der Fokus wurde hierbei auf sieben Forschergruppen [11, 13–16, 19, 25] gelegt, welche an insgesamt *n* = 213 Patienten den zusätzlichen Effekt einer präoperativen PNE zu einer biomedizinischen Edukation (*n* = 114) im Vergleich zu einer alleinigen präoperativen biomedizinischen Edukation (*n* = 99) bei Patienten vor Operationen an verschiedenen Körperbereichen untersuchten. Zwei Untersuchungen [10, 26] verglichen an insgesamt 147 Patienten vor einer Wirbelsäulenoperation weitere Formen präoperativer Aufklärung (Führung durch die Klinikräumlichkeiten, zusätzlich schriftliches Material über das OP-Prozedere, detailliertere Erklärungen des OP-Prozederes etc.).

Sechs der eingeschlossenen Studien weisen ein sehr gutes Evidenzlevel (1 bzw. 1B) auf [10, 11, 13, 16, 25, 26]. Drei Studien haben eine geringere Evidenz (3B und 4; [14, 15, 19]).

Aus den eingeschlossenen Studien geht hervor, dass bislang kein standardisiertes Vorgehen einer präoperativen PNE existiert. Ein Großteil der Autoren bezieht sich auf das Vorgehen der Arbeitsgruppen von Louw et al. Nahezu keine Angaben machen die Autoren für den genauen Zeitpunkt der präoperativen PNE. Lediglich von einer Forschungsgruppe wird angegeben, dass die edukativen Einheiten in den Wochen 8, 7, 6 und 5 vor der OP stattfinden sollen [11]. Louw et al. [14] führen die präoperative PNE 28 bis 2 Tage vor der OP durch. Nach Louw et al. [13] ist der beste Zeitpunkt einer präoperativen Edukation eine Woche vor der OP.

Eine größere Konsistenz besteht zwischen den Autoren für die Art der Informationsvermittlung und das Setting.

Alle Autoren, welche eine PNE durchführten, wählten ein auditiv-visuelles Vorgehen [11, 13–15, 19, 25]. Außer von Núñez-Cortés et al. [25] wurde dieses durch schriftliches Material untermauert. Das gewählte Setting war bei allen Untersuchungen, welche die positiven Aspekte einer Kleingruppe in den Vordergrund stellten, im 1:1-Kontakt.

Weniger Konsistenz besteht für die Dauer und Anzahl der präoperativen PNE, wobei ein Großteil aller Autoren eine Einheit von 30 min durchführte [13–15, 19, 25]. Eine Forschungsgruppe führte vier Einheiten mit einer Dauer von 20 bis 60 min im Abstand von einer Woche durch [11]. Lediglich bei einer Erhebung wurde eine präoperative PNE mit einer postoperativen Eigenübungstherapie kombiniert [25] und eine weitere Untersuchung kombinierte eine präoperative Edukation mit präoperativer Mobilisation [11].

Im Durchschnitt erhielten die Patienten eine präoperative PNE-Einheit von 35 min.

Zusammenfassend herrscht Uneinigkeit hinsichtlich der Art, Dauer und des Settings. Überwiegend wurde eine einmalige 30-minütige visuell-auditive Edukation mit zusätzlichem schriftlichem Material im 1:1-Kontakt durchgeführt. Über den Zeitpunkt der Edukation kann aufgrund der großen Inkonsistenz keine Aussage getroffen werden. Von den meisten Autoren wurde ein zeitlicher Abstand zwischen der Edukation und der OP von mehr als einem Tag gewählt.

Die Autoren untersuchten v. a. die Domänen Schmerz, Funktionseinschränkung, Katastrophisierung, Kinesiophobie sowie die postoperative Inanspruchnahme von Leistungen im Gesundheitswesen.

Die Ergebnisse zeigen Tendenzen auf, wonach eine präoperative PNE v. a. die psychometrischen Variablen Katastrophisierung (kein Effekt (*n* = 43) [19, 25]; signifikanter Effekt (*n* = 55) [11, 14, 15]). Kinesiophobie (kein Effekt [25]; signifikanter Effekt [11, 19]) sowie die Sensitivität des Nervensystems im betroffenen Körpergebiet (kein Effekt [11]; signifikanter Effekt [15, 19]) positiv beeinflusst. Keinen signifikanten Effekt hat eine präoperative PNE auf den postoperativen Schmerz und Funktion. Eine Intensivierung der biomedizinischen Aufklärung kann die postoperative Angst steigern [26]. Wie die Follow-up-Erhebungen zeigten [13, 16], scheint eine postoperative Verhaltensveränderung im Sinne einer signifikant geringeren erneuten Inanspruchnahme von Leistungen im Gesundheitswesen nicht in Zusammenhang mit Schmerz und Funktion zu stehen, sondern eher mit den psychometrischen Variablen Katastrophisierung und Bewegungsangst.

Eine Übersicht der eingeschlossenen Artikel gibt Tab. 4 mit den entsprechenden Abkürzungen in Tab. 5.

**Table 4:** 

Artikel, Erscheinungsjahr	Studiendesign, Evidenzlevel	Probanden	1. Messkriterien, 1a. primäre Messkriterien, 1b. sekundäre Messkriterien	Messinstrumente	Ergebnisse	Schlussfolgerung
			2. Messzeitpunkte			
			3. Intervention			
**Núñez-Cortés et al. (2019)** [25]	Doppelt verblindete RCT, 1B	Pat. vor einer CTS-OP IG: *n* = 16, CG: *n* = 15	**1.** Schmerz Funktion der oberen Extremität (OE) Katastrophisieren Kinesiophobie Angst‑/Depression	VAS QuickDASH („short form of the Disabilities of Arm, Shoulder and Hand Questionnaire“) PCS Tampa Scale of Kinesiophobia(TSK)-11 HADS	Schmerz: – Kein sig. Gruppenunterschied zu jedem Zeitpunkt – Sig. Unterschied für beide Gruppen für Baseline – 4./12. W. postop. Funktion der OE: – Kein sig. Gruppenunterschied zu jedem Zeitpunkt – Sig. Unterschied für beide Gruppen für Baseline – 4./12. W. postop. Katastrophisieren: – Keine sig. Gruppenunterschiede zu den Zeitpunkten 4. und 12. W. postop. – Sig. Gruppenunterschied präop. Kinesiophobie – Kein sig. Gruppenunterschied zu den Zeitpunkten 4. + 12. W. postop. – Sig. Gruppenunterschied zum Zeitpunkt präop.	Beide Interventionen haben einen sig. Einfluss auf den postoperativen Schmerz und die Funktion der OE, aber nicht auf die psychometrischen Variablen Katastrophisierung und Kinesiophobie
			**2.** – Präop. (keine genauere Angabe) – Baseline – 4. Woche postop. – 12. Woche postop.			
			**3.** Präoperative auditiv-visuelle 1:1-Edukation + Anleitung von postoperativen Übungen. Keine exakte Angabe zum genauen Zeitpunkt der Edukation *IG:* 30 min PNE: neurophysiologische und biopsychosoziale Aspekte von Schmerz, periphere und zentrale Sensibilisierung + 7. Tag postop. Übungsanleitung + schriftliche Heimübungen *CG:* 30 min Standardedukationseinheit: Infos zu medizinischen, anatomischen und pathologischen Aspekten des CTS + 7. Tag postop. Übungsanleitung + schriftliche Heimübungen			
**Lee et al. (2018)** [10]	RCT, 1B	82 Patienten mit bevorstehender Wirbelsäulen-OP Interventionsgruppe (IG): *n* = 42 Kontrollgruppe (KG): *n* = 40	**1a**. Angst, Schmerz	State-Trait Anxiety Inventory (STAI) – „20-items state construct items“ „Visual analog scale of pain“ (VAS) Patientenmonitor (HP M1205A; Philips Medical Systems, MA) Saliva (2470 Wizard2 Automatic Gamma Counter; PerkinElmer, Fremont, CA)	Tag vor OP: keine sig. Gruppenunterschiede für Angst, Schmerz, physische Indikatoren 30 min vor OP: sig. reduzierte Angst und Schmerz in IG, keine sig. Gruppenunterschiede bzgl. der physischen Indikatoren Tag nach OP: sig. reduzierte Angst und Schmerz in IG, keine sig. Gruppenunterschiede bzgl. der physischen Indikatoren, aber reduzierter Puls und RR in IG Vgl. der Messzeitpkt.: *IG:* Angst: 1 > 2 > 3 Schmerz: 2 > 1 > 3 *KG:* Angst: 2 > 1 > 3 Schmerz: 2 > 1 = 3	Präoperative intensive Edukation reduziert die Angst prä- und postoperativ sowie den Schmerz postoperativ
			**1b.** Physische Indikatoren: Puls, Atemfrequenz, Blutdruck, Kortisollevel			
			**2.** 1. M.: Tag vor OP, 2. M.: 30 min vor OP, 3. M.: Tag nach OP			
			**3.** Edukative Intervention präop. *IG:* 5‑ bis 10-minütige verbale Standardinformationen zum Operationsprozedere, Beantwortung der Patientenfragen. Zusätzliche 20-seitige Informationsbroschüre über den genauen OP-Ablauf + erklärende Bilder und Videos *KG:* 5- bis 10-minütige verbale Standardinformationen zum Operationsprozedere, Beantwortung der Patientenfragen			
**Lluch et al. (2018)** [11]	RCT, 2‑armig, „parallel group, assessor-blinded“, 1B	Pat. mit Knieschmerzen aufgrund einer Osteoarthritis >3 Monate, vor einer TEP-OP IG: *n* = 22 CG: *n* = 22	**1a.** CPM (objektiver Biomarker von CS)	NRS WOMAC CSI PCS TSK-11 CPM durch QST: – PPT – TS *1. PPT:* – „analog Fisher algometer (Force Dial model FDK 40), rate of 1 kg/cm^2^/s“ – Je 3 Messungen mit einer Pause von 30 s – 3 cm medial des medialen Patellarands – 3 cm lateral des lateralen Patellarands – Ipsilateral: 5 cm distal des lateralen Epicondylus humeri (auf M. extensor carpi radialis longus) *2. TS:* – 10 konsekutiv ansteigende Impulse an jeder PPT-Teststelle (2kg/s - 4 - 6 - 8 - 10 - 12 - 14 - 16 - 18 - 20kg/s) 5 min Pause *3. CPM:* Kombination aus TS und einem „conditioning stimulus“ (schmerzhafte Reize am kontralateralen Arm durch aufgeblasene Manschette). Die Manschette wurde mit 20 mm Hg/s aufgeblasen, bis ein erster Schmerz auftrat, und dies für 30 s gehalten. Schmerz wurde dann erhoben. Nach 30 s wurde der Druck der Manschette so verändert, dass der Schmerz mit 3/10 angegeben wurde. Dann wurde TS wiederholt	Schmerz und Funktion verbesserte sich sig. für alle Messzeitpunkte ohne Gruppenunterschiede CPM: – Messlokalisationen: sig. geringer am lat. Knie und Epikondylus im Vgl. zum med. Knie – Interaktion zwischen Gruppe und Zeit: einzige sig. Veränderung für die IG zwischen Baseline und 3 Monate postop. – Keine sig. Veränderungen für CG TS: – Keine Unterschiede bzgl. der Lokalisation und zwischen den Gruppen – Für beide Gruppen keine Unterschiede zwischen den Messzeitpunkten PPT: – Sig. Unterschiede bzgl. Lokalisation: lat. Knie > med. Knie, lat. Knie > Epikondylus – Keine sig. Unterschiede zwischen den Gruppen – Sig. Zeitunterschiede in beiden Gruppen: sig. höhere PPT zu jedem Zeitpunkt im Vgl. zu Baseline CSI: – Sig. Zeitunterschiede in beiden Gruppen ohne Gruppenunterschiede für die Messzeitpunkte: a) 3 M. postop. – Baseline, b) 3 M. postop. – direkt nach Edukation, c) 3 M. postop. – direkt präop. WOMAC: – Zeitliche Reduktion des Scores in beiden Gruppen für: a) 3 M. postop. – Baseline, b) 3 M. postop. – direkt nach Edukation, c) 3 M. postop. – direkt präop. PCS: – Interaktion zwischen Gruppe und Zeit: – IG: sig. Reduktion aller Messzeitpunkte im Vgl. zu Baseline – CG: sig. Reduktion für direkt präop. – Baseline und direkt präop. – direkt nach Edukation, kein sig. Unterschied für Baseline – 3 M. postop. – Sig. Gruppenunterschiede zu den Zeitpunkten: direkt nach Edukation und 3 M. postop. (IG sig. besser)	Durch eine präoperative PNE + Kniemobilisation konnte im Vgl. zu einer präoperativen biomedizinischen Edukation + Kniemobilisation eine sig. Verbesserung in den Parametern Katastrophisierung und Kinesiophobie sowie CPM erreicht werden Keine sig. Gruppenunterschiede liegen für die Parameter Schmerz, Funktion, zentrale Sensibilisierung, temporale Summation und PPT vor Sig. Zeitunterschiede in beiden Gruppen für die Parameter Schmerz, Funktion, zentrale Sensibilisierung und PPT
			**1b.** PPT TS Schmerz Funktion Zentrale Sensibilisierung Katastrophisierung Kinesiophobie			
			**2.** – 1. Baseline (2 M. präop.) – 2. Sofort nach 4 präop. Einheiten (1 M. präop.) – 3. 1 Monat Follow-up (direkt präop.) – 4. 3 Monate postop.			
			**3.** *IG:* PNE gefolgt von „knee joint mobilization“, Buch „Schmerzen verstehen“ *CG:* biomedizinische Edukation gefolgt von „knee joint mobilization“ (Probanden sind verblindet hinsichtlich der Art der Edukation) 4 edukative Einheiten präop., je 1×/W. in Woche 8, 7, 6, 5 vor OP 1. Einheit: 50–60 min 2.–4. Einheit: 20–30 min Edukation: verbal und visuell mit PC, schriftlich („Your nerves are having knee replacement“; Louw 2012) *Inhalte:* PNE: Physiologie des Nervensystems, akuter vs. chronischer Schmerz, wie wird Schmerz chronisch, CS, Rekonzeptualisierung von postoperativem Schmerz nach TEP Biomedizinisch: Anatomie und Biomechanik des Knies, Ätiologie, Symptome, Behandlungen, OP-Prozedere Mobilisation mit Bewegung nach Mulligan (3 × 10 Wdh.)			
					TSK-11: – Keine sig. Unterschiede für CG – Sig. Zeitunterschiede für IG: a) Baseline – direkt nach Edukation, b) Baseline – direkt präop., c) Baseline – 3 M. postop., d) 3 M. postop. – direkt nach Edukation – Sig. Gruppenunterschiede zu den Zeitpunkten: a) direkt nach Edukation, b) direkt präop., c) 3 M. postop.	
**Louw et al. (2014)** [13]	Multicenter-RCT, 1B	65 Pat. mit lumbaler Radikulopathie vor OP IG: *n* = 31 KG: *n* = 34	**1a.** – Rücken‑/Beinschmerz – Funktion	NPRS ODI Fragebogen bzgl. der Erfahrungen des Pat. mit OP und präoperativer Edukation Eigene Angaben des Pat. zu Häufigkeit von postoperativen, medizinischen Untersuchungen und ärztlichen u./o. therapeutischen Therapien	Rücken‑/Beinschmerz: zu keinem der Messzeitpkt. sig. Unterschied zwischen den Gruppen. Beide Gruppen hatten sig. weniger Schmerz von Baseline zu 1 Monat postop. Funktion: zu keinem der Messzeitpkt. sig. Unterschied zwischen den Gruppen In 3/5 Pkt. zeigte sich die IG sig. zufriedener mit OP und präoperativer Edukation Die postoperativen Kosten durch die Inanspruchnahme medizinischer/therapeutischer, postoperativer Leistungen war bei der EG sig. geringer (45 %) (v. a. Röntgen und physikalische Therapie in KG sig. höher)	Keine sig. Gruppenunterschiede für Schmerz und Funktion. Beide Gruppen erreichten eine sig. Schmerzreduktion und Funktionsverbesserung Sig. postoperative Verhaltensveränderungen sowie eine starke Reduktion der eigenen Inanspruchnahme von Leistungen im Gesundheitswesen konnten bei Patienten erzielt werden, welche präoperativ eine neurobiologische Edukation bekamen
			**1b.** – Gedanken des Pat. bzgl. OP – Inanspruchnahme von postoperativen Leistungen im Gesundheitswesen			
			**2.** – Baseline (vor Edukation) – 1 Monat postop. – 3 Monate postop. – 6 Monate postop. – 12 Monate postop.			
			**3.** *IG:* biomedizinische OP-Vorbereitung + PNE (30 min verbale Edukation im 1:1-Kontakt + Pat. erhält eine Informationsbroschüre mit den Inhalten der PNE, welche mind. 1 × vor und 1 × nach der OP gelesen werden soll) *KG:* biomedizinische OP-Vorbereitung			
**Louw et al. (2016)** [16]	RCT, 1B	50 Patienten mit 1‑Jahres-Follow-up nach Dekompressions-OP Davon waren *n* = 22 in der IG und *n* = 28 in der CG	**1a.** Postoperative Inanspruchnahme von Leistungen im Gesundheitswesen	Utilization of Healthcare Questionnaire NPRS ODI: „A change of 5 points (10 %) is reported to be the MDC (minimal detectable change) for LBP populations“ Fragebogen bzgl. individueller Gedanken und Erfahrungen mit der OP und präoperativen Edukation	Keine weitere „healthcare utilization“: 10 IG, 6 KG Weitere LWS-OP im Zeitraum zwischen 1 Jahr und 3 Jahre postop.: 1 IG, 3 KG Ausgaben für Medikamente: 37 % geringer in IG (nicht sig.) Kosten für Arzt/Therapeut im Zeitraum von der OP bis 3 Jahre postop.: KG sig. höher für Hausarzt, physikalische Therapie, Massage Zum Zeitpunkt 3 Jahre postop. keine Unterschiede der Kosten in allen Bereichen LWS- und Beinschmerz, Funktion: keine sig. Gruppenunterschiede zum Zeitpunkt 3 Jahre postop. Zufriedenheit: Gruppenunterschiede: EG war sig. zufriedener für die Punkte: „ich fühlte mich umfassend vorbereitet“, „ich fühlte mich gut vorbereitet“, „meine Erwartungen wurden erfüllt“. Im zeitlichen Vgl. keine sig. Unterschiede in EG und KG	Trotz nicht-sig. Unterschiede bzgl. Rücken- und Beinschmerz sowie subjektiv empfundener Einschränkung (Funktion) im 3‑Jahres-Follow-up hat die IG sig. seltener Leistungen im Gesundheitswesen in Anspruch genommen im Zeitraum bis 3 Jahre postop. sowie sich seltener aufgrund von Schmerzen erneut operieren lassen
			**1b.** Rücken- und Beinschmerz, Funktion, OP-Zufriedenheit			
			**2.** 3 Jahre postop.			
			**3.** *IG:* präoperative verbale edukative Standardvorbereitung + PNE mittels eines 1:1-Gesprächs *KG:* präoperative verbale edukative Standardvorbereitung			
**Rhodes et al. (2015)** [26]	Prospektive, randomisierte Studie, 1B	65 Jugendliche mit adoleszenter idiopathischer Skoliose und ihre Eltern IG: *n* = 26 KG: *n* = 39	**1a.** Angst der Patienten	State-Trait Anxiety Inventory Child (STAI-C) State-Trait Anxiety Inventory (STAI) Aufenthaltsdauer in Tagen Medikamente in mg/g/kg Fragebogen des KH bzgl. Zufriedenheit von Pat. + Eltern	Angst der Patienten: – 2 W. präop.: IG = KG – Direkt präop.: IG = KG – 2 T. postop.: IG sig. höher KG – Entlassung: IG sig. höher KG Zu allen Zeitpkt. zeigten beide Gruppen sig. höhere „state anxiety“ als „trait anxiety“ Angst der Eltern: keine sig. Unterschiede zw. den Gruppen und im zeitl. Verlauf In beiden Gruppen reduzierten sich die State-anxiety-Werte im zeitl. Verlauf, die Trait-anxiety-Werte blieben stabil Aufenthaltsdauer und Medikamenteneinnahme: keine sig. Gruppenunterschiede Zufriedenheit: Pat.: IG > KG (sig.) Eltern: IG > KG (nicht sig.)	Eine intensive präoperative Edukation das OP-Prozedere betreffend sowie ein präoperatives Kennenlernen relevanter postoperativer Aufenthaltsbereiche des Krankenhauses hat keinen Effekt auf die Angst der Jugendlichen und deren Eltern, auf die Aufenthaltsdauer und die Einnahme der Medikamente. Eine Entwicklung einer präoperativen entängstigenden Edukation für Patienten und Eltern ist wichtig, um die postoperative Angst zu reduzieren
			**1b.** – Angst der Eltern – Länge des KH-Aufenthalts – Schmerzmedikamente – Zufriedenheit der Patienten und ihrer Eltern			
			**2.** – 2 Wochen präop. – Direkt präop. – 2 Tage postop. – Tag der Entlassung			
			**3.** *IG:* – Präoperatives Gespräch über Risiken, „benefit“ und Alternativen der geplanten OP – 30-min-Führung durch KH (präoperativer Bereich, Intensivstation, Folgestation, Familienzimmer, Therapieräume) – Genaue Beschreibung des OP-Prozederes *KG:* – Präoperatives Gespräch über Risiken, „benefit“ und Alternativen der geplanten OP			
**Louw et al. (2015)** [15]	Fall-Kontroll-Studie, 3B	30-jährige Patientin mit Rücken- und Beinschmerz li. > re., akut seit ca. 3 Monaten	**1.** – Angst-Vermeidungs-Verhalten – Schmerzkatastrophisierungsgedanken – Funktion – Rücken- und Beinschmerz – Kenntnisse über die Neurophysiologie des Schmerzes – Rumpfflexion – Nervengleitfähigkeit – Schmerzschwelle – Hirnaktivitäten	FABQ PCS ODI VAS NPQ Fragebogen bzgl. der Gedanken und Erwartungen die OP betreffend FBA (1×) SLR (2×) (Inklinometer) PPT (MW aus 3 Messungen) – Dominante Hand – Li. neben Proc. spinosus von L3 – Li. Kniekehle fMRI-Scanner	FABQ, NPQ: kein Effekt PCS: 10 Pkt. reduziert ODI: 10 % reduziert Positivere Gedanken und Erwartungen die OP betreffend FBA: 8 cm weiter SLR: 7° höher PPT: reduziert fMRI: 1. Deaktivierung des periaquäduktalen Graus 2. Deaktivierung des Cerebellums 3. gesteigerte Aktivität des Motorkortex	Die sofort sichtbaren positiven Veränderungen im fMRI sowie die Veränderungen der psychometrischen Erhebungen und funktionellen Testungen weisen darauf hin, dass eine präoperative PNE einen Effekt auf kortikaler Ebene hat bei Patienten vor einer Operation der lumbalen Wirbelsäule
			**2.** – Vor der präoperativen PNE – Nach der präoperativen PNE			
			**3.** Präoperative PNE 1:1-Setting 30 min			
**Louw et al. (2020)** [19]	„Case series“, 4	12 Patienten mit Schulterschmerz und eingeschränkter Schulterbeweglichkeit, vor einer Schulter-OP	**1.** – Schmerz – Schmerzkatastrophisierung – Bewegungsangst – Eigene Gedanken zur Schulter-OP – Zentrale Sensibilisierung – Aktive ROM in Flexion der betroffenen Schulter – Sensitivität des Nervensystems:a) Deltoidansatz (betroffene Schulter) b) Deltoidansatz (nichtbetroffene Schulter) c) Posteriore Kniegelenksfalte der betroffenen Seite	NPRS PCS TSK 6 Fragen zu eigenen Gedanken bzgl. der anstehenden OP („10-point Likert scale“) CSI Goniometer PPT (angegeben werden sollte der Moment, in dem der Druck übergeht zu Schmerz) „Mean“ von 3 aufeinanderfolgenden Messungen (→ 15 % Veränderung = signifikant)	Schmerz: kein sig. Unterschied (*p* = 0,125) Schmerzkatastrophisierung: Reduktion von 1,5 Pkt., nicht statistisch sig., unterhalb des MDC Kinesiophobie: sig. Reduktion von 4 Pkt. (*p* = 0,013), aber ohne klinische Relevanz, da der MDC bei 5,6 Punkten liegt (33,3 % der Pat. erreichten eine Veränderung > MDC) Gedanken der Pat.: positive, aber nicht-sig. Veränderungen aller Gedanken Aktive Flex.: statistisch sig. Vergrößerung der ROM (*p* = 0,013), aber ohne klinische Relevanz, da Veränderung unterhalb des MDC CSI: keine Angaben Sensitivität des Nervensystems: Betroffene Schulter: sig. und klinisch relevante Verbesserung. Nichtbetroffene Schulter: nicht-sig. Verbesserung. Knie: nicht-sig. Verbesserung	PNE hat keinen sig. Einfluss auf Schmerz und Katastrophisierung. PNE hat einen sig. Einfluss auf Bewegungsangst und die aktive Beweglichkeit der Schulter, aber ohne klinische Relevanz PNE reduziert sig. die Sensitivität des Nervensystems im Bereich der betroffenen Schulter
			**2.** Direkt vor und direkt nach der PNE			
			**3.** 1:1-PNE 30 min, adaptiert für Pat. vor einer Schulter-OP, in Anlehnung an PNE-Programme für Pat. vor einer LWS- oder Knie-OP + schriftliches Material. Keine exakte Angabe zum genauen Zeitpunkt der Edukation			
**Louw et al. (2015)** [14]	Fallbeschreibungen, 4	10 Patienten (7 w, 3 m)	**1.** – Rücken- und Beinschmerz – Funktion – Angst-Vermeidung: a) körperliche Aktivität, b) Arbeit/Job – Katastrophisieren (den Schmerz und die Verletzung betreffend) – Kenntnisse über die Neurophysiologie des Schmerzes – Rumpfflexion – Nervengleit- und -spannungsfähigkeit	NPRS ODI FABQ – „Physical activity“ (FABQ-PA) – „Work“ (FABQ-W) PCS NPQ FBA „Passive straight leg raise“ ([P]SLR) – Inklinometer	Rückenschmerz: – Direkt nach Edukation: 6/10 höherer Schmerz, 1/10 MDC – 1 Monat postop.: 8/10 geringerer Schmerz (6/10 MDC) – 3 + 6 Monate postop.: 9/10 NRS ≤2 Beinschmerz: – Direkt nach Edukation: 7/10 hatten höheren Schmerz – 1, 3, 6 Monate postop.: Die meisten hatten keinen Schmerz FABQ-PA: – Direkt nach Edukation: Reduktion – 1, 3, 6 Monate postop.: Reduktion des Scores um 50 % FABQ-W: – Direkt nach Edukation: Score um 25 % geringer PCS: – Direkt nach Edukation: 10/10 hatten geringere Katastrophisierungsgedanken (5/10 MDC) – 1, 3, 6 Monate postop.: 8/10 im Bereich des MDC NPQ: – Zu jedem Messzeitpkt. bei 10/10 Pat. 10 % besser ODI: – Direkt nach Edukation: 7/10 MDC – 6 Monate postop.: 6/10 20 % geringer FBA: – Direkt nach Edukation: 6/10 Pat. im Bereich des MDC oder mehr SLR: – Direkt nach Edukation: 6/10 Pat. im Bereich des MDC oder besser	Eine neurobiologische Edukation hat einen direkten Einfluss auf den Patienten. Deutliche Unterschiede konnten bzgl. der Katastrophisierungsgedanken, der Rumpfflexion, der Nervengleitfähigkeit sowie der eigenen Gedanken des Pat. die bevorstehende OP betreffend festgestellt werden. Die Studie zeigt keinen direkten positiven Effekt auf die empfundene Schmerzstärke, wobei diese Veränderungen außerhalb des MDC liegen und deshalb infrage gestellt werden müssen. Allgemein ist die Aussagekraft der Studie aufgrund des geringen Evidenzlevels als gering einzustufen
			**2.** – Baseline (vor Edukation) – Direkt nach der Edukation – 1 Monat postop. – 3 Monate postop. – 6 Monate postop.			
			**3.** Neurobiologische Edukation 30 min im 1:1-Kontakt präop. + Buch („Your nerves are having back surgery“)

**Table 5:** 

CPM	„Conditioned pain modulation“
CSI	„Central sensitization inventory“
CTS	Karpaltunnelsyndrom
DASH	Disability of Arm, Shoulder and Hand Questionnaire
FABQ	Fear Avoidance Belief Questionnaire
FBA	Finger-Boden-Abstand
HADS	Hospital Anxiety and Disability Questionnaire
IG	Interventionsgruppe
KG	Kontrollgruppe
LBP	„Low back pain“
MDC	„Minimal detectable change“
NPQ	Neurophysiology of Pain Questionnaire
NPRS	„Numeric pain rating scale“
NRS	„Numeric rating scale“
ODI	Oswestry Disability Index
OE	Obere Extremität
PCS	„Pain catastrophizing scale“
PNE	„Pain neuroscience education“
PPT	„Pain pressure threshold“
QST	Quantitative sensorische Testung
SLR	„Straight leg raise“
STAI	State-Trait Anxiety Inventory
TEP	Totalendoprothese
TS	Temporale Summation
TSK	Tampa Scale of Kinesiophobia
VAS	Visuelle Analogskala
WOMAC	Western Ontario and McMaster Universities Osteoarthritis Index

*RCT* Randomized Controlled Trial, *CG* Control group, *IG* Interventriosngruppe, *fMRT* funktionelle Magnetresonanztomografie

## Diskussion

Für die Überprüfung der Validität der Primärstudien spielt die Untersuchung eines möglichen Bias eine wichtige Rolle. Wichtige Parameter sind in diesem Zusammenhang die untersuchte Population sowie Verblindungs- und Randomisierungsprozesse. Die Validität der Primärstudien wird überwiegend als gut bezeichnet (gute Validität [10, 11, 13, 16, 25]; mäßige Validität [19]; geringe Validität [14, 15]). Die Generalisierbarkeit der Ergebnisse auf weitere Populationen ist eher gering (hohe Generalisierbarkeit [10]; geringe Generalisierbarkeit [11, 13–16, 19, 25, 26]). Kritisch zu betrachten sind die Ergebnisse v. a. deshalb, da es sich in einigen Studien um sehr kleine Populationsgruppen handelt bzw. z. T. eine Kontrollgruppe fehlt.

In Bezug auf die *primäre Forschungsfrage* besteht der Wert der Studien v. a. darin, dass Tendenzen aufgezeigt werden konnten, welche eine positive Beeinflussung des postoperativen Outcomes durch eine edukative Intervention vor einer Operation belegen. Diese zeichnen sich v. a. durch eine Veränderung der psychometrischen Variablen (Kinesiophobie und Katastrophisierung) sowie durch eine reduzierte Sensitivität des Nervensystems aus. Keine Veränderungen zeigten sich hinsichtlich Schmerzintensität und Funktion. Dass trotzdem eine Verhaltensveränderung im Sinne einer signifikant geringeren Inanspruchnahme von postoperativen Leistungen im Gesundheitswesen zu beobachten ist, zeigt eindeutig, dass Patienten nicht aufgrund der alleinigen Schmerzintensität oder der eingeschränkten Funktion nach externer Hilfe suchen, sondern auch aufgrund der damit verbundenen Ängste und Sorgen, welche u. a. durch Schmerzen ausgelöst werden können. Kritisch anzumerken ist an dieser Stelle, dass von den meisten Autoren diese Ergebnisse nur maximal in einem 3‑Monats-Follow-up bestätigt wurden, über langfristigere Outcomes sind bislang nur wenige Ergebnisse bekannt [13, 14, 16]. Ein 12- und 24-Monats-Follow-up ist in dem Studienprotokoll von Ickmans et al. (2016) vorgesehen [8], welches aufgrund der Einschlusskriterien nicht in die Ergebnisauswertung aufgenommen wurde.

Bezüglich der *sekundären Forschungsfrage* zeigen die Studien eine größere Inkonsistenz in der Herangehensweise der präoperativen PNE. Zwar überschneiden sich größtenteils die Inhalte der präoperativen PNE, allerdings liegen Variationen in Zeitpunkt und Dauer vor. Einigkeit herrscht hinsichtlich eines visuell-auditiven Settings mit unterstützendem schriftlichen Material. Welchen Umfang das schriftliche Material hat, ob hierbei viele Bilder oder Verlinkungen zu weiteren Informationsquellen geboten werden, bleibt meist unklar. Auch wurde die präoperative Edukation überwiegend im 1:1-Kontakt durchgeführt. Die positiven Aspekte einer Gruppentherapie, welche im nichtoperativen Setting zu guten Ergebnissen führten, wurden von den Autoren nicht beleuchtet. Bislang gibt es keine Untersuchungen zu den Effekten einer präoperativen PNE im Gruppensetting. Ob der von den Forschern gewählten Herangehensweise im 1:1-Kontakt ein übergeordneter Effekt zugeschrieben werden kann, kann nicht beantwortet werden.

Obwohl in dem bereits in 2. Auflage erschienenen Leitfaden „Pain Neuroscience Education“ von Louw et al. (2018) [17] eindeutig eine Kombination aus PNE und Übungstherapie empfohlen wird, führten lediglich zwei Autoren [11, 25] eine solche kombinierte Herangehensweise durch. Auch hier liegen somit Unstimmigkeiten in der Herangehensweise vor.

Zusammenfassend sind die ausgewählten Primärstudien v. a. für die Beantwortung der primären Forschungsfrage von großer Bedeutung. Sie zeigen einheitlich die Tendenz auf, dass eine präoperative PNE zu einer Verbesserung psychometrischer Variablen führt und somit das postoperative Outcome nicht durch die Parameter „Schmerz“ und „Funktion“ bestimmt wird. In Bezug auf die sekundäre Forschungsfrage legen sie in der gemeinsamen Betrachtung Unklarheiten hinsichtlich einer evidenzbasierten Herangehensweise offen.

Ein möglicher Bias kann gegebenenfalls dadurch entstehen, dass fünf der neun eingeschlossenen Studien von Louw et al. durchgeführt wurden.

### Mögliche neurobiologische Mechanismen der positiven Effekte von PNE

Eine Vielzahl von kortikalen Regionen, welche für das Verhalten, Emotionen, Erfahrungen etc. verantwortlich sind, ist während einer Schmerzwahrnehmung gleichzeitig aktiv und durch Nervenzellen miteinander verknüpft. Dieses sich permanent verändernde Netzwerk von Nervenzellen wird Schmerzneuromatrix oder Konnektom genannt [9, 23, 30]. Dysfunktionen im zentralen und/oder peripheren Nervensystem unterhalten die Aktivitäten in der kortikalen Matrix und können zu nicht zielführenden Copingstrategien führen [5].

Die fMRT-Untersuchungen von Louw et al. [15] zeigten nach der PNE eine Deaktivierung in Arealen des periaquäduktalen Graus (PAG) und des Cerebellums sowie eine gesteigerte Aktivität im Motorkortex. Besonders dem PAG und dem Cerebellum wird eine wichtige Rolle in der absteigenden Schmerzmodulation, dem Vermeidungsverhalten sowie dem subjektiven Empfinden einer Bedrohung zugeschrieben. Das PAG steht über direkte Bahnen mit dem Gyrus cinguli in Verbindung, welcher an der emotionalen Bewertung von Schmerz beteiligt ist [1, 4]. Wie lange die gefundenen kortikalen Veränderungen bei der untersuchten Patientin bestehen blieben, ist aufgrund fehlender weiterer Messungen unklar. Wichtig erscheint, dass diese Ergebnisse aufgrund des Einzelfalldesigns nicht generalisierbar sind.

Die durch eine Schmerzerfahrung hervorgerufenen kortikalen neurobiologischen Veränderungen im Sinne einer zentralen Sensibilisierung stehen in einem engen Zusammenhang mit den psychometrischen Charakteristika Kinesiophobie und Katastrophisieren [22]. Diese enge Verknüpfung von Schmerz und Emotionen wird auch in einem Review von Lumley et al. (2011; [21]) beleuchtet.

Um die Entwicklung kinesiophober und katastrophisierender Verhaltensweisen zu verhindern oder deren weitere Ausprägung zu reduzieren, ist es fundamental, den Schmerz sowohl bei akuten als auch bei chronischen Schmerzsyndromen für seine Bewältigung zu verstehen [3, 5].

Die Rekonzeptualisierung von Schmerz und einer Schmerzerfahrung steht im Hauptfokus der PNE.

Zahlreiche Autoren vertreten die Meinung, dass das postoperative Outcome bereits durch die präoperative Phase mit beeinflusst werden kann [11, 13–15, 19].

Auf Basis der Ergebnisübersicht kann hypothetisiert werden, dass eine präoperative neurobiologische Erklärung von Schmerz im postoperativen Stadium die Sensitivität des Nervensystems sowie kinesiophobe und schmerzkatastrophisierende Tendenzen reduziert. Diese Ergebnisse konnten unabhängig von der Lokalisation des OP-Bereichs bestätigt werden.

Durch Langzeit-Follow-up-Erhebungen konnte eine Verhaltensveränderung im Sinne einer geringeren Inanspruchnahme von postoperativen Leistungen im Gesundheitswesen beobachtet werden.

Keinen Einfluss hat eine präoperative PNE auf die postoperative Schmerzintensität und Funktion.

Hinsichtlich der Herangehensweisen liegen v. a. in Zeitpunkt und Dauer der präoperativen PNE Inkonsistenzen vor. Um den Effekt der präoperativen PNE zu stärken sowie ihren Nutzen weiter zu optimieren, besteht hier weiterer Forschungsbedarf.

Die Analyse der Herangehensweise von PNE (sowohl bei akuten und chronischen Schmerzen als auch im präoperativen Kontakt) im klinischen Setting offenbart die Notwendigkeit einer intensiven Erklärung von Schmerz. Dass der empfundene Schmerz immer eine Leistung des Gehirns ist, sollte unabhängig von körperlicher Diagnose und Heilungsstadium der Struktur immer Teil eines aufklärenden Gesprächs aller Berufsgruppen sein. Die Erläuterung einer Funktionsstörung, welche alleinig für einen Schmerz verantwortlich gemacht wird, reicht für keinen Schmerzphänotyp aus, um die Bedrohung eines Schmerzes zu reduzieren.

Kritisch ist zu betrachten, dass die durchgeführten präoperativen PNE-Interventionen ausschließlich im 1:1-Setting durchgeführt wurden, was im klinischen Alltag nahezu nicht umsetzbar erscheint. Welchen Effekt eine Gruppenintervention hat, ist bislang nur aus schmerztherapeutischen Interventionen im Rahmen der Behandlung eines chronischen Schmerzes bekannt. V. a. die positiven sozialen Aspekte einer Gruppentherapie können von der Autorin der vorliegenden Arbeit durch Erfahrungen im klinischen Alltag bestätigt werden. Vor dem Gesichtspunkt eines biopsychosozialen Ansatzes wäre eine präoperative PNE im Gruppensetting somit ein sinnvoller Ansatz [6].

## Fazit für die Praxis

- „Pain neuroscience education“ (PNE) in einem präoperativen Setting kann Kinesiophobie, Angst und Katastrophisieren reduzieren. Der Effekt einer präoperativen PNE kann weniger an den Variablen Schmerz und Funktion bewertet werden, sondern eher durch psychometrische Assessments wie z. B. TSK und PCS.
  Die Inhalte von PNE basieren auf der Neurobiologie und Ätiologie von Schmerzen sowie von zu Schmerz beitragenden Risikofaktoren.
- PNE hat einen systematischen Aufbau und kann einfach in die tägliche Praxis integriert werden.
